# Progressive right ventricular dilatation after repair of tetralogy of Fallot: myth or reality? A single center evaluation by repeat cardiovascular magnetic resonance over 37 months

**DOI:** 10.1186/1532-429X-17-S1-P223

**Published:** 2015-02-03

**Authors:** Tobias Rutz, Susanne Naumann, Christian Meierhofer, Stefan Martinoff, Peter Ewert, Heiko Stern, Sohrab Fratz

**Affiliations:** Service de Cardiologie, Centre Hospitalier Universitaire Laudois, Lausanne, Switzerland; Deutsches Herzzentrum München, Munich, Germany

## Background

Surgical repair of tetralogy of Fallot (TOF) frequently leads to pulmonary regurgitation and right ventricular (RV) dilatation. This study assesses the rate of progression of RV dilatation over time and the impact of surgical correction with and without transannular patch repair.

## Methods

Fifty-one patients underwent two cardiac magnetic resonance (CMR) exams (time between exams 37±21 months) with determination of RV and left ventricular (LV) volumes and pulmonary regurgitant fraction (PR). Three groups with different repair techniques were examined: transannular patch repair (TA, n=22), subvalvular patch repair (SV, n=15) or infundibulectomy (IN, n=14).

## Results

No patient had RV outflow tract obstruction or undergone RV outflow tract intervention. TA patients were significantly younger: TA 17±10 vs. SV 22±9 vs. IN 28±11 years, p=0.005. RV enddiastolic volume index (RVEDVI) and PR did not change significantly in the whole group: RVEDVI: 118±23 vs. 119±23 ml/m^2^, p=0.684. PR: 32±11 vs. 32±11%, p=0.772. There was no significant difference of RVEDVI in each group between first and last CMR: TA 120±21 vs. 122±22 ml/m^2^, p=0.516; SV 112±23 vs. 111±23ml/m^2^, p=0.700; IN 123±28 vs. 123±25ml/m^2^, p=0.936.

RVEDVI at last CMR and change of RVEDVI per year did not differ between groups: TA 122±22 vs. SV 111±23 vs. IN 123±25ml/m^2^, p=0.301; change RVEDVI: TA 0.4±9.3 vs. SV -0.4±7.3 vs. IN -2.2±13.1ml/m^2^/y, p=0.742.

## Conclusions

There is no significant progression of RV dilatation in patients after TOF repair with moderately dilated RV during a median follow-up of 37 months. Valve sparing repair techniques, however, do not to preclude from RV dilatation.

**Figure 1:**
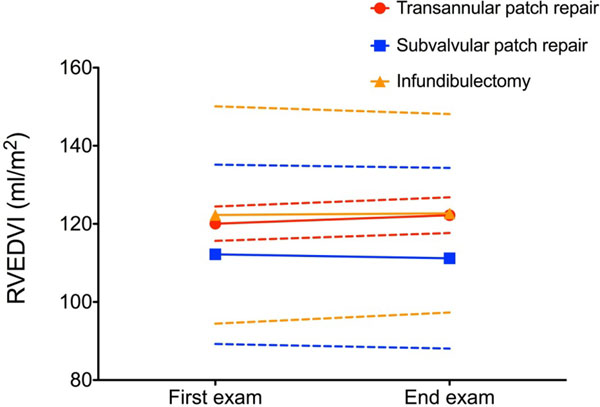
RVEDVI = right ventricular enddiastolic volume index

